# Probiotic and Potentially Probiotic Yeasts—Characteristics and Food Application

**DOI:** 10.3390/foods10061306

**Published:** 2021-06-07

**Authors:** Adam Staniszewski, Monika Kordowska-Wiater

**Affiliations:** Department of Biotechnology, Microbiology and Human Nutrition, University of Life Sciences in Lublin, Skromna 8, 20-704 Lublin, Poland; adam.staniszewski@up.lublin.pl

**Keywords:** probiotics, probiotic yeasts, *Saccharomyces cerevisiae* var. *boulardii*, potentially probiotic yeasts

## Abstract

Probiotics are live microorganisms which when administered in adequate amounts confer a health benefit on the host. Besides the well-known and tested lactic acid bacteria, yeasts may also be probiotics. The subject of probiotic and potentially probiotic yeasts has been developing and arising potential for new probiotic products with novel properties, which are not offered by bacteria-based probiotics available on the current market. The paper reviews the first probiotic yeast *Saccharomyces cerevisiae* var. *boulardii*, its characteristics, pro-healthy activities and application in functional food production. This species offers such abilities as improving digestion of certain food ingredients, antimicrobial activities and even therapeutic properties. Besides *Saccharomyces cerevisiae* var. *boulardii,* on this background, novel yeasts with potentially probiotic features are presented. They have been intensively investigated for the last decade and some species have been observed to possess probiotic characteristics and abilities. There are yeasts from the genera *Debaryomyces*, *Hanseniaspora*, *Pichia*, *Meyerozyma*, *Torulaspora*, etc. isolated from food and environmental habitats. These potentially probiotic yeasts can be used for production of various fermented foods, enhancing its nutritional and sensory properties. Because of the intensively developing research on probiotic yeasts in the coming years, we can expect many discoveries and possibly even evolution in the segment of probiotics available on the market.

## 1. Introduction

According to the World Health Organization (WHO) and the Food and Agriculture Organization of the United Nations (FAO), probiotics are “live microorganisms which when administered in adequate amounts confer a health benefit on the host” [1]. Health benefits have been predominantly demonstrated for specific probiotic strains of the bacteria genera *Lactobacillus*, *Bifidobacterium*, *Enterococcus*, *Streptococcus*, *Pediococcus, Leuconostoc*, *Bacillus* and *Escherichia* [2], while the only yeast genus that has been proven effective in double-blind studies is *Saccharomyces* [3].

**Figure 1:**
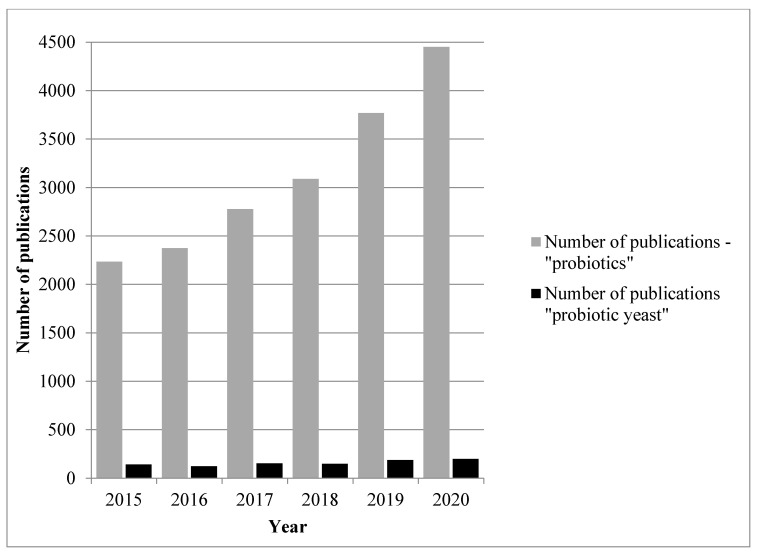
Number of PubMed publications under the terms “probiotics” and “probiotic yeast” in the last 5 years.

Probiotics are able to grow at 37 °C, survive unfavourable conditions of human digestive track (e.g., digestive enzymes, pancreatic juice and low pH) and contribute to the health of the host environment by regulating microbiota as well as exerting biological functions; some also adhere to gut epithelial cells’ mucus [2]. Within the last years, the interest in this subject has increased; PubMed indexes over 31,000 articles that use the term probiotic and over 15,000 have been published within the last five years (Figure 1), but probiotic yeast research is a minor part of this with fewer than 850 articles indexed by PubMed within the last 5 years (Figure 1). The aim of the publication is to review the latest information about probiotic and potentially probiotic yeasts and their application in various kinds of functional food.

## 2. Properties of Perfect Probiotic Strain

Screening for promising probiotic candidates could be time-consuming and expensive, but certain properties have proven helpful and allow carrying out initial in vitro screening. These properties fall under two groups—functional and technological features. In functional features, we can distinguish four main properties: ability to survive delivery to the target organ, interaction with host systems, antipathogenic properties and safety. Most probiotics are taken orally to reach the intestinal tract (GI tract) as its target organ. Thus, they must survive transit from the mouth to the GI tract. This implies screening potential probiotic strains for resistance to environmental conditions inside the GI track (digestive enzymes, gastric and bile salts, pH and body temperature of host), ability to colonise mucosal surfaces and ability to withstand the gut’s microbiota (auto- and coaggregation capability, surface hydrophobicity and antibiotic resistance) [4]. The selected strain must also be species identified, strain typed and tested by means of safety (strain cannot produce toxins, be pathogenic or have hazardous metabolic activities) and must be able to survive the manufacturing process [5]. Technological features include an ability to easily produce large amounts of biomass, resistance to preservation procedures such as lyophilisation with high shelf life in the finished product, genetic stability and no deterioration of the organoleptic characteristics of final products [6,7].

## 3. *Saccharomyces cerevisiae* var. *boulardii*

The history of probiotic yeast dates back to the early 20th century, when Henri Boulard isolated the original strain from fruits in Indochina [8]. Since the 1950s, it has been widely used as a commercially available treatment for diarrhoea worldwide. The taxonomic position of *S. cerevisiae* var. *boulardii* is debatable [9,10], but current literature and Index Fungorum claim *S. cerevisiae* var. *boulardii* to be no more than a variety of *S. cerevisiae*, closely related to the *S. cerevisiae* wine strains [11,12]. *S. cerevisiae* var. *boulardii* was originally described as a separate species—*Saccharomyces boulardii*—but rapid development of molecular phylogenetics in recent years has led to a change in its classification, as has happened with many yeast species, and it is currently classified as *Saccharomyces cerevisiae* var. *boulardii*. Despite having some unique properties, it cannot be regarded as a distinct species [13,14]. According to McFarland [10], there are some important differences between *S. cerevisiae* var. *boulardii* and *S. cerevisiae* at the physiological (i.e., lack of ability to use galactose as carbon source and lack of ability to produce ascospores) and molecular levels (i.e., individual chromosome and gene copy numbers). This was confirmed by Edwards-Ingram et al. [13]. The main differences between these microorganisms are presented in Table 1.

The results published by Mitterdorfer et al. [14] show that either *Saccharomyces cerevisiae* or *Saccharomyces cerevisiae* var. *boulardii* amplification product (1170 bp) specific for *S. cerevisiae* could be obtained during species-specific polymerase chain reaction with primers SC1/SC2 [15]. Nevertheless, they showed that distinctive fingerprint patterns for *S. cerevisiae* var. *boulardii* could be produced via RAPD; in addition, restriction profiles of the ITS region with four endonucleases applied (MseI, MspI, ScrFI and TaqI) were identical for all *S. cerevisiae* var. *boulardii* strains and always differed from all of the others. 

Edwards-Ingram et al. [16] reported that *S. cerevisiae* var. *boulardii* is a strain of *S. cerevisiae* that has lost most of its Ty1/2 elements, while data obtained by Khatri et al., show the presence of Ty2 elements but absence of Ty1, Ty3 and Ty4 elements [11]. However, how important these distinct properties of *S. cerevisiae* var. *boulardii* are for its probiotic potency is not fully known yet. Comparative transcriptome analysis conducted by Pais et al., presents significant differences in expression levels of various genes between *S. cerevisiae* var. *boulardii* and *S. cerevisiae* under GI-track-like conditions. They also suggested 30 genes which are predicted to be associated with the main probiotic properties of *S. cerevisiae* var. *boulardii* including genes associated with poliamine metabolism, carbon source assimilation and acetate production [17]. The list of genes mentioned by Pais et al., is presented in Table 2. Moreover, there are genes with higher copy number in *S. cerevisiae* var. *boulardii* than in *S. cerevisiae* responsible for protein synthesis (*RPL31A, RPL41A, RPS24B, RPL2B* and *RSA3*) and stress response (*HSP26, SSA3, SED1, HSP42, HSP78* and *PBS2*). It is possible that these genes support increased growth rate, pseudo-hyphal switching and higher resistance to high pH. Duplicated and triplicated genes mostly encode stress response proteins, elongation factors, ribosomal proteins, kinases, transporters and fluoride export, which may be helpful in adaptation to stress conditions. *S. cerevisiae* var. *boulardii* is also reported to have several genes with different number of copies related to pseudo-hyphal growth (*CDC42, DFG16, RGS2, CYR1, CDC25, STE11, SKM1* and *RAS1*). The higher maximum number of repetitive sequences within flocculation genes (e.g., *FLO1*), which may affect adhesion and flocculation ability, was also identified in *S. cerevisiae* var. *boulardii* [17].

Multiple mechanisms (modulation of normal microbiome of the gut, antagonism against pathogens, adhesion to the mucus, immune modulation and trophic effects on GI tract) have been proposed for the probiotic action of *S. cerevisiae* var. *boulardii* [18,19]. *S. cerevisiae* var. *boulardii* helps to restore the normal microbiota of the gut in patients after antibiotic therapy or surgery and may temporarily work as a replacement of the natural microbiome until it is re-established. Among various modes of antimicrobial activity, there are secretion of special proteins that cleave microbial toxins (i.e., cholera toxin) or reduce cAMP levels responsible for diarrhoea and the ability to inhibit *Escherichia coli* surface endotoxins by dephosphorylation. Other mechanisms include stimulation of immunoglobulin A production against *Clostridium difficile* toxin A, degradation of the toxin by a secreted protease [20,21,22] and modulation of cytokine production [23]. *S. cerevisiae* var. *boulardii* could preserve enterocyte barrier integrity by stimulating tight junction protein secretion and could reduce or exclude pathogens from interaction with intestinal epithelial cells by binding directly to the pathogen cells via mannose residues in the yeast cell wall [20]. Secretion of antimicrobial compounds in the form of peptides, hydrogen peroxide and organic acids features prominently among the generally accepted action mechanisms of bacterial probiotics, but none of the direct inhibitory actions on bacterial growth or antimicrobial compound secretion by this species has been reported [24]. Trophic effects postulated for *S. cerevisiae* var. *boulardii* are also a very interesting subject. Among the effects, it is especially worth highlighting such effects as stimulation of brush border membrane; secretion of digestive enzymes, e.g., sucrase-iso-maltase, maltase-glucoamylase, lactase-phlorizin hydrolase, alanine aminopeptidase, alkaline phosphatase and nutrient transporters (sodium-glucose transport proteins), which may be induced by polyamines; and modulation of short- and branched-chain fatty acids synthesis, which play various roles in the physiological and biochemical functions in different tissues (intestine, liver, adipose, muscle and brain) [19].

Several studies have been conducted using *S. cerevisiae* var. *boulardii* in the treatment of gastrointestinal diseases such as foodborne and traveller’s diarrhoeas; Crohn’s and inflammatory bowel disease; irritable bowel syndrome; adults and children’s acute gastroenteritis; and HIV-infected chronic diarrhoea caused by *Clostridium difficile*, *Vibrio cholerae* and other pathogenic enterobacteria. In addition, research conducted by Profir et al., shows significant reduction in the intensity of toxocariasis [3,25,26,27,28]. Additionally, probiotic yeasts have been used to reduce side effects of treatments against *Helicobacter pylori* [28,29]. The efficiency of probiotic yeasts has been documented in several clinical studies [3,30,31,32,33,34]. Das et al., showed in a randomised clinical trial that the dose of 250 mg twice a day for children under 5 years old significantly shortened the diarrheal duration and duration of hospitalisation without any adverse events, but it had no effect on the duration of fever or vomiting in acute rotavirus diarrhoea in children [33]. Feizizadeh et al., based on a meta-analysis of 22 randomised control trials, concluded that *S. cerevisiae* var. *boulardii* might be effective in treating acute childhood diarrhoea regardless of its causes and can significantly decrease stool frequency and the risk ratio of diarrhoea in children. The studies included in the meta-analysis did not show any major side effects related to *S. cerevisiae* var. *boulardii*, but these trials were carried out on previously healthy children, excluding malnutrition and immunodeficient patients [31]. For those groups, data are limited, but some case studies occur. Thygesen et al., described a case report of 79-year-old woman who developed *S. cerevisiae* var. *boulardii* fungemia (SCF) after bowel resection [35]. Kara et al., described two cases of SCF after probiotic treatment of intensive care unit patients [36]. Ellouze et al., reported cases of septic shock after *S. cerevisiae* var. *boulardii* treatment [37]. SCF has also been reported in patients with *Clostridium difficile*-associated diarrhoea who have been treated orally with *S. cerevisiae* var. *boulardii* in association with antibiotic treatment [38]. However, most cases concern severely ill or immunocompromised patients.

## 4. Novel Strains of Yeast with Probiotic Potential

Within the last years, interest in the subject of novel yeast with potentially probiotic properties has been increasing. Novel isolates have been isolated from diverse products and environments such as fruit and vegetables, fermented food and beverages, industrial dairy wastes, etc. Novel isolates must have all properties required for probiotics strain, fulfil safety requirements and have good manufacturing properties. Isolation of various species from numerous environments allows discovering new probiotic strains with innovative biochemical properties, for example the ability to extracellularly secret lactase which may confer the additional ability to digest whey used as food additive in animal feed. Recent studies report evidence that in addition to *S. cerevisiae* var. *boulardii* other species have probiotic properties, e.g., *Kluyveromyces marxianus* and *Pichia kudriavzevii*. The European Food Safety Authority (EFSA) has granted the QPS status (qualified presumption of safety) to only a few yeasts which might be used as “food additive”, i.e., *K. marxianus* var. *lactis* and *K. marxianus* var. *fragilis* [39]. Several studies conducted on non-*Saccharomyces* yeasts demonstrated the presence of probiotic potential. Ochangco et al., investigated *Debaryomyces hansenii* strains obtained from cheese and fish guts. During the research, they selected strain DI 02 as the best probiotic candidate because of its outstanding ability to survive the GI stresses, adhere to Caco-2 cells and mucin and induce higher anti-inflammatory response than *S. cerevisiae* var. *boulardii* (the authors used the anti-inflammatory cytokine IL-10 to pro-inflammatory cytokine IL-12 ratio as an indicator of anti-inflammatory properties). The other strain, DI 09, adhered more strongly to Caco-2 cells and mucin. Two strains (DI 10 and DI 15) induced a higher IL-10/IL-12 ratio than the *S. cerevisiae* var. *boulardii* strains, indicating higher anti-inflammatory effects on human dendritic cells [40]. The results obtained by Oliveira et al., suggest that some yeasts isolated from fermented table olives such as *Pichia guilliermondii* 25A and *Candida norvegica* 7A have probiotic potential because of their resistance to the simulated digestive track’s conditions on a similar level as *S. cerevisiae* var. *boulardii*, the reference strain used in the research [41]. Gil-Rodrigues et al., analysed 130 yeast strains from a culture collection and observed that two strains of *Schizosaccharomyces pombe* (IFI-936 and IFI-2180) display a high capacity to thrive in the host intestine (good growth at 37 °C, good tolerance to GI tract conditions and high autoaggregation percentage) and a high antioxidant activity [42]. From the 108 identified yeasts strains of various origin, Rodríguez et al., showed that two yeasts, *Hanseniaspora osmophila* and *P. kudriavzevii*, were the most promising strains on the basis of statistical analyses applied in each step of selection [43]. All scientists highlight that future studies are needed for the final selection including the GRASS character of the selected strains. Table 3 presents a summary of the data on the novel strains described and their sources of origin.

## 5. Probiotic and Potentially Probiotic Yeasts in the Aspect of Functional Food

The term “functional food” is usually used as a marketing term with various definition and it is not recognised by law globally. The exception is Japan, where the law treats functional foods as a separate food category. According to the International Food Information Council (IFIC), functional foods are “foods or dietary components that may provide health benefit beyond basic nutrition” [51,52].

Probiotics due their properties beneficially affect various physiologic functions, which allow them to be classified as functional foods [53]. In the last years, various studies including the use of probiotic and potentially probiotic yeasts in food have been published. Senkarcinova et al., showed the possibility of using a probiotic strain of *S. cerevisiae* var. *boulardii* in production of low-alcohol and alcohol-free beer [54]. The data published by Ramirez-Cota et al., also suggest the ability of the species to survive ethanol concentration occurring in the most popular craft beer styles; thus, it is potentially possible to create probiotic-fortified beer [55]. Mulero-Cerezo et al., reported that “*Saccharomyces cerevisiae* var. *boulardii* as a single yeast starter produces craft beer with higher antioxidant activity, lower alcohol content, similar sensory attributes and higher yeast viability after 45 days than that produced by a commercial *Saccharomyces cerevisiae* strain commonly used in the brewery industry” [56]. The results published by de Paula et al., also show that functional beer containing *S. cerevisiae* var. *boulardii* after storage and in vitro GI transit had a population of living cells above the minimal dose prescribed for health benefit [57].

Probiotic yeast could be used not only for beverages, but various other products as well. Swieca et al., suggested the use of *S. cerevisiae* var. *boulardii* as a food additive to enrich bean sprouts and use them as a carrier for probiotics. This additive did not affect any properties of the sprouts, and the yeast significantly improved microbiological quality of the final products [58]. Sarwar et al., developed symbiotic yogurt with *S. cerevisiae* var. *boulardii* and inulin. The combination of yeast and inulin increased amount of favourable volatile compounds and improved product texture in comparison to plain, control yogurt [59]. Yeasts and lactic acid bacteria (LAB) are often isolated together from various spontaneously fermented foods [60,61,62,63,64,65,66,67,68]. Karaolis et al., investigated the potential application of *S. cerevisiae* var. *boulardii* as a probiotic in goat’s yoghurt with lactic acid bacteria starter cultures. The authors indicated that *S. cerevisiae* var. *boulardii* promoted the growth of LAB, and its concentration was steady during the whole storage period [69]. Similar mutually stimulating interactions between *S. cerevisiae* and LAB occur in sourdough fermentation [70]. Xu et al., described interaction between *Lactobacillus* and *Saccharomyces cerevisiae*. The interaction is complex and dependent on the composition and production process of fermented foods. Usually, the relationship between LAB and yeast is mutualistic for both groups of microorganisms; however, this does not always mean a positive effect on the final product. For example, malolactic fermentation carried out by *Lactobacillus* in wine and beer might be desirable and beneficial in some types of beverages such as sour beers, but, in most cases, acidification is seen as a product defect, often caused by contamination during production process [71,72,73]. *S. cerevisiae* secretes several growth factor such as carbon dioxide and amino acids which encourage *Lactobacillus* growth; the release of carbon dioxide provides a local micro-anaerobic environment preferred by *Lactobacillus spp*. [74]. The yeasts also secrete amino acids such as threonine, glutamine, alanine, glutamate, serine and glycine, promoting the growth of LAB and allowing LAB to grow in environments which otherwise would not be possible [75]. In fermented milk products, *Lactobacillus* decomposes lactose (the main sugar in milk foods, which *S. cerevisiae* cannot metabolise) into galactose, providing carbon sources for yeasts. Next to galactose, lactic acid produced by LAB might also be used as a carbon source under aerobic condition, while the assimilation of the lactic acid under this condition might stimulate specific species of *Lactobacillus* to produce higher amounts of kefiran—a food-derived biopolymer with potential for use within food and biomedical applications [68,70,76,77,78,79]. Moreover, probiotic and potentially probiotic yeast can be used in fermentation of grain products. The consumption of whole, multigrain grain products has many advantages, but whole grain products present many antinutrients. Banik et al., reported the ability to use probiotic *S. cerevisiae* APK1 starter cultures as biofortification of multigrain substrates used as a base in traditional Indian dishes. The fermented product showed significant improvement in the increment of protein, fibre and starch content and decreasing the level of antinutrients. Furthermore, during fermentation, antioxidant potential and the level of total phenolic and total flavonoid contents increased [80]. Besides, probiotic *Saccharomyces* has an interesting beneficial effect on the nutritional value of foods of plant origin since it synthesises folates and eliminates phytates and other antinutrients. Enzymes—phytases produced by this yeast—enhance the bioavailability and absorption of essential minerals such as iron, zinc, magnesium and phosphorus [81]. Another advantage of *S. cerevisiae* var. *boulardii* may be its antimicrobial properties and ability to decompose mycotoxins such as aflatoxins, patulin, ochratoxin A and others [82,83]. Naimah et al., reported that antimicrobial peptides isolated from *S. cerevisiae* var. *boulardii* inhibit growth of *Bacillus cereus*, *Escherichia coli*, *Pseudomonas aeruginosa* and *Staphylococcus aureus* [84]. Goktas et al., also reported antimicrobial activity against *Salmonella* Typhimurium, *Yersinia enterocolitica*, *Candida albicans*, *Alternaria alternata* and *Aspergillus flavus* in strains of *S. cerevisiae* var. *boulardii* isolated from commercial food supplements [85].

Besides *S. cerevisiae* var. *boulardii* usage in production of novel functional products, the probiotic strain *Pichia kudriavzevii* OG23 was used by Ogunremi et al., to produce fermented, cereal-based food. They reported increased antioxidant activity and a variety of flavour compounds. They also suggested the ability to use cereal-based products as delivery vehicle for probiotics [86]. Amorim et al., compared *S. cerevisiae* var. *boulardii* and *Meyerozyma caribbica* for pineapple beverage production and the beverage properties. The results reveal that two strains of *M. caribbica*, isolated from pineapple’s skin, showed desirable in vitro probiotic properties similar to the reference probiotic strain *S. cerevisiae* var. *boulardii*. Strain 9D of *M. caribbica* was selected to be used in a fermentation study. The obtained beverage had high antioxidant activity, and the data show that the antioxidant activity was not affected by the fermentation process. The beverage produced with 9D strain also had good sensorial characteristics and was well accepted by consumers, compared to the beverage obtained by fermentation with *S. cerevisiae* var. *boulardii* [48]. Table 4 shows examples of novel probiotic and potentially probiotic strains for potential application in food.

## 6. Maintaining the Viability of Probiotic Yeasts in Food

A minimum dose of 10^6^ colony forming units per millilitre or gram (CFU/mL or CFU/g) must be reached for the food product to be labelled as probiotic [87]. As viability of microorganism is the key to achieve the health benefits, some researches even suggest increasing the dose up to 10^7^ CFU/mL or CFU/g [88,89,90]. There are several ways to accomplish the goal which depends on environmental conditions in final product and its interaction with the probiotic strain. The food matrix’s chemical composition and its physical state affect and can interrupt growth, stability and survival of probiotic microorganisms during product storage and GI transition [91]. From the technological point of view, it is favourable if microbial cultures are capable of growing in substrate media, survive during processing and maintain their viability throughout the storage. If the product’s matrix provides this condition, the dosage of probiotic microorganism during production can be reduced due to self-propagation of the microorganism [92]. Otherwise, if the environmental condition within the matrix does not allow proliferation of the probiotic strain, experimental dose determination is required, or usage of other methods which will increase strain survivability might be necessary [92]. The most commonly used methods of protective strategies are encapsulation (cells are closed in protective shells made of food grade polymers such as chitosan, gelatine or alginate [90,93]), addition of protective agents (i.e., cryoprotectants and osmoprotectants) and usage of miscellaneous carriers [94,95,96,97]. Microencapsulation of *S. cerevisiae* var. *boulardii* has been reported many times. These yeast’s cells were entrapped in sodium alginate beads to protect them from adverse conditions [98,99,100]. Scientists confirmed that microencapsulation assured yeast survival and its controlled release. The encapsulation of *S. cerevisiae* var. *boulardii* with a mixture of alginate, inulin and mucilage was also used to design new functional products such as cheeses and yogurts, and it increased the viability of yeast and extended the full benefits of the product compared with the product supplemented with free or non-encapsulated cells [101]. Arslan et al., (2015) found that the use of gelatin and arabic gum for *S. cerevisiae* var. *boulardii* microencapsulation at higher temperatures resulted in yeast with higher resistance to simulated gastric processes.

Bevilaqua et al., (2020) investigated the effect of microencapsulation into alginate gels on the functional properties of probiotic yeasts and confirmed that yeasts in beads did not affect such properties as hydrophobicity, autoaggregation and biofilm formation. On the other hand, encapsulation affected protection of the cells against simulated GI conditions. Finally, the kinetic study showed that alginate beads may be useful as reusable carriers of starter cultures or probiotics into the gut [100].

## 7. Conclusions

Research on probiotics has been dynamically developing in recent years, including the use of probiotic yeasts, which has been minimised thus far, and is gaining more and more interest. The latest research shows the wide potential of the use of probiotic yeast in the food industry and the use of their unique properties thus far not found in probiotic bacteria. The most known probiotic yeast, *S. cerevisiae* var. *boulardii,* has been investigated in detail, and many of its characteristics concerning beneficial effects on human health and the positive or negative influence on food matrices have been reported. Besides *S. cerevisiae* var. *boulardii,* there are other yeasts with potential probiotic activity, but they need to be investigated because the information about them is very scarce. These yeasts (from genera *Pichia*, *Hanseniaspora*, *Torulaspora*, *Metchnikowia,* etc.), which are isolated from food and non-food habitats, are the objects of intensive studies nowadays, and there is a real chance to introduce them into various kinds of food not only for fermentation processes but also for supplementation as valuable nutrients with health benefits. The coming years will bring more information and possibly also a wider use of probiotic yeast in food.

## Figures and Tables

**Table 1:** Differences between Saccharomyces cerevisiae and Saccharomyces cerevisiae var. boulardii.

	*Saccharomyces cerevisiae*	*Saccharomyces cerevisiae* var. *boulardii*
Ability to grow at 37 °C	-	+
Usage of galactose as carbon source	+	-
Ability to produce ascospores	+	-
Ability to survive pH 2.5	-	+
Additional copies IX chromosome	-	+
Enhanced ability for pseudohyphal switching	-	+
Ploidy	diploid or haploid	diploid

**Table 2:** Genes predicted to be associated with the main probiotic properties of *S. cerevisiae* var. *boulardii* (data acc. to Pais et al. [17]).

Poliamine Metabolism	Carbon Source Assimilation	Acetate Production
AGP2	CYC8	ACS2
ARG7	GAL1	ADH1
CAR2	GAL7	ALD4
PTK1	IMA1	ALD5
TPO1	MIG1	CIT3
TPO2	PGM1	IDP3
TPO4	SUC2	LSC2
	TUP1	MAE1
		MDH3
		MLS1
		PDC6
		SDH2
		SDH5
		SHH3
		SHH4

**Table 3:** Novel potentially probiotic strains of yeast.

Species	Strain	Origin	References
*Candida orthopsilosis*	CCMA 1748	Naturally fermented table olives, Brazil	[44]
*Candida tropicalis*	CCMA 1751	Naturally fermented table olives, Brazil	[44]
*Debaryomyces hansenii*	CCMA 1761	Naturally fermented table olives, Brazil	[44]
	DI02	Dairy isolate, Denmark	[40]
*Hanseniaspora osmophila*	1056, 1094	Food environment, YBL of the UCLM, Spain	[45]
*Kluyveromyces marxianus*	B0399	Whey, BCCM (accession number MUCL 41579)	[46]
*Lachancea thermotolerans*	B13	Moss on oak, Italy	[47]
*Meyerozyma caribbica*	9D	Pineapple, Brazil	[48]
	CCMA 1758	Naturally fermented table olives, Brazil	[44]
*Metschnikowia ziziphicola*	B27	Beech tree bark, Italy	[47]
*Pichia fermentans*	BY5	Raw milk, China	[49]
*Pichia guilliermondii*	CCMA 1753	Naturally fermented table olives, Brazil	[44]
*Pichia kudriavzevii*	BY10, BY 15	Raw milk, China	[49]
*Saccharomyces cerevisiae*	3, 146	Food environment, YBL of the UCLM, Spain	[45]
	6, 7, 8, 10c, 2PV	Verdicchio wine, Italy	[47]
	AKP1	Haria (traditional Indian food), India	[50]
	CCMA 1746	Naturally fermented table olives, Brazil	[44]
*Torulaspora delbrueckii*	35, 1.1t2, 7.3t2, c7.4, j401, tdvcsff	Sugar cane juice, Cameroon	[47]
*Yarrowia lipolytica*	HY4	Raw milk, China	[49]

Abbreviations: YBL of the UCLM, Culture Collection of the Yeast Biotechnology Laboratory of the University of Castilla-LaMancha; BCCM, Belgian Coordinated Collection of Microorganisms.

**Table 4:** Novel probiotic and potentially probiotic strains of yeast for potential application in food.

Strains	Product	Added Value ^1^	References
*Pichia fermentans* BY5	-	Cholesterol reduction	[49]
*Pichia kudriavzevii* BY10 *Pichia kudriavzevii* BY15	- -	Cholesterol reduction	[49]
		Cholesterol reduction	
*Meyerozyma caribbica* 9D	Fermented pineapple beverage	Better sensory properties with lower ethanol content	[48]
*Saccharomyces cerevisiae* var. *boulardii* CNCM I-745 *Saccharomyces cerevisiae* var. *boulardii* (strain from Biopron Forte)	Craft beer	Possibility to produce functional beer with high ethanol concentration	[55]
	Low-alcohol and alcohol-free beer	Production of alcohol-free and low-alcohol products	[54]
*Yarrowia lipolytica* HY4	-	Cholesterol reduction	[49]

^1^ An aspect in which the use of the strain shown can bring innovative properties to the product or improve its properties.
